# Endometriosis and adverse pregnancy outcomes: A case-control study

**DOI:** 10.18502/ijrm.v22i6.16798

**Published:** 2024-08-05

**Authors:** Fatemeh Shahmoradi, Ladan Haghighi, Marziyeh Noori, Roya Derakhshan, Neda Hashemi, Samaneh Rokhgireh

**Affiliations:** ^1^Department of Obstetrics and Gynecology, Iran University of Medical Sciences, Tehran, Iran.; ^2^Endometriosis Research Center, Iran University of Medical Sciences, Tehran, Iran.; ^3^Department of Artificial Intelligence, Smart University of Medical Sciences, Tehran, Iran.

**Keywords:** Endometriosis, Pregnancy outcomes, Pre-eclampsia, Preterm birth, Cesarean section, Small for gestational age.

## Abstract

**Background:**

The association between endometriosis and the outcome of pregnancy is one of the interesting topics. Endometriosis-related pain is alleviated with pregnancy; however, it is known to cause adverse outcomes in pregnancy. The main cause is systemic chronic inflammation caused by higher levels of cytokines, growth factors, and angiogenesis factors.

**Objective:**

This study aimed to clarify the relationship between endometriosis, deep endometriosis, adenomyosis, surgical treatment, and poor maternal consequences.

**Materials and Methods:**

In this case-control study, data from 250 women who gave birth in Hazrat Rasoul Akram hospital, Tehran, Iran from February 2015 to December 2019 was extracted from the hospital information system in January 2020. Participants were divided into 2 groups: 125 women with endometriosis and 125 women without endometriosis. We looked at how endometriosis affected mothers and newborn babies. Data on pregnancy, delivery, and newborns of both groups was extracted.

**Results:**

The mean age of participants was 32.74 ± 4.10 and 31.7 ± 5.53 yr in endometriosis and control group, respectively. In terms of pregnancy complications, placenta previa, placenta accreta, placenta abruption, pre-eclampsia, gestational diabetes mellitus, and postpartum hemorrhage remarkably increased in the endometriosis group compared to the control group. Small for gestational age was significantly higher in rectal endometriosis than women without rectal endometriosis (p = 0.03). The neonatal intensive care unit admission rate was notably higher in infants of the endometriosis group compared to controls (40.7% vs. 24.8%, p = 0.009).

**Conclusion:**

Our findings showed women with endometriosis are at a higher risk for important adverse maternal outcomes.

## 1. Introduction

Endometriosis is a debilitating disease. Its prevalence is 10–15% in reproductive age and 30–50% in women with infertility (1–4). Endometriosis has 3 main features: superficial peritoneal lesions, ovarian endometrioma, and deep endometriosis. Common epigenetic changes in endometriosis and adverse pregnancy outcomes are documented in the literature. The main complaints of women with endometriosis are dysmenorrhea, dyspareunia, dyschezia, chronic pelvic pain, and infertility. Adenomyosis is related to endometriosis and is presented by infertility, menometrorrhagia, and pain (5, 6).

In recent decades, the documented relationship between endometriosis and increased risk of obstetrical complications such as preterm birth, hypertensive disorders of pregnancy, gestational diabetes mellitus (GDM), small for gestational age (SGA), placenta previa, placental abruption, placenta accrete, and postpartum hemorrhage (PPH) has been the focus of research (7–12).

Meanwhile, deep endometriosis has attracted the attention of many researchers. A considerable relationship between rectovaginal endometriosis and placenta previa has been found (13). However, there is still no evidence that severe endometriosis can be associated with an increased risk of poor obstetric outcomes. Moreover, there is no evidence of the effect of endometriosis surgery on reducing obstetric complications or aggravating complications during pregnancy (14, 15).

On the other hand, due to infertility caused by endometriosis, many women need assisted reproductive methods for fertility, and they are at increased risk for obstetric complications of pregnancy (16, 17). The underlying mechanisms of adverse obstetric consequences in endometriosis are poorly understood. Common epigenetic changes in endometriosis and poor outcomes of pregnancy may be responsible for obstetric complications in women with endometriosis (18).

Since the effect of endometriosis on pregnancy outcomes is not well elucidated, this study aimed to clarify the relationship between endometriosis, deep endometriosis, adenomyosis, surgical treatment, and poor maternal consequences. The present article has been uploaded to the preprint site of (research square).

## 2. Materials and Methods

In this case-control study, data from 250 women, who gave birth in Hazrat Rasoul Akram hospital (a referral center for endometriosis and high-risk pregnancies), Tehran, Iran from February 2015 to December 2019 were extracted from the hospital information system and endometriosis data registry of the endometriosis research center of Iran University of Medical Sciences, Tehran, Iran.

Participants were divided into 2 groups: 125 women with endometriosis and 125 women without endometriosis. We also had a subgroup of rectal endometriosis in endometriosis group. We looked at how endometriosis affected mothers and newborn babies.

### Sample size

The sample size was calculated using the above formula of 150 people in each group. Ultimately, the information of 125 people from each group was analyzed. 


$$N={\left(Z_{1-\alpha }+Z_{1-\beta }\right)}^{2}\left(P_{1}\left(1-P_{1}\right)\right)+\left(P_{2}\left(1-P_{2}\right)\right)/D^{2}$$

α = 0.05, ß = 0.2, p1 = 12.3, p2 = 5.2

### Inclusion and exclusion criteria 

The 2 groups of endometriosis and control included women aged 15–45 yr with and without endometriosis, respectively. Diagnosis of endometriosis was confirmed through laparoscopy with histological confirmation in the endometriosis group; all 125 women in the endometriosis group underwent surgery for endometriosis, with 68 of them having rectal endometriosis involvement.

The control group (125 women) who were referred to the prenatal clinic had no prior history of endometriosis based on clinical and imaging evaluations before pregnancy.

Exclusion criteria were gestational age of < 22 wk, fetal malformations, twin pregnancies, insufficient medical records, underlying diseases such as overt diabetes, cardiac diseases, hypertension, neurology and psychological disorders, and autoimmune diseases. We adjusted for maternal age to prevent the effect of increasing pregnancy complications with the increasing age of the mother.

### Outcomes

Comprehensive questionnaires regarding pregnancy, childbirth, and neonatal details were completed for both groups, with additional information sourced from medical records. Maternal and neonatal characteristics were manually extracted from the electronic medical record system; however, endometriosis data were obtained from the endometriosis data registry of the endometriosis research center of Iran University of Medical Sciences, Tehran, Iran.

Maternal outcomes included GDM, pre-eclampsia, gestational hypertension, PPH described as bleeding more than 500 ml after delivery, obstetric complications such as abortion, ectopic pregnancy, placenta previa, placental abruption, placenta accreta, delivery mode (cesarean section [CS]), normal vaginal delivery. Infant outcomes, such as preterm birth ( < 37 wk), SGA, neonatal intensive care unit (NICU) admission, and mortality, were also documented.

### Ethical considerations

The Ethical Committee of Iran University of Medical Sciences, Tehran, Iran, approved this study (Code: IR.IUMS.FMD.REC.1398.422). The data of participants is confidential with the corresponding author.

### Statistical analysis

For the analysis, participants' demographic characteristics have been presented as mean ± SD, obstetrics and neonatal characteristics were compared using the Chi-squared test for the categorical variables, and the unpaired *t* test or Mann-Whitney U tests for the continuous variables according to normal or non-normal distributions. The *t* test or Wilcoxon signed-rank test was used to compare dependent variables at different times.

Logistic regression analysis was used to investigate the relationship between endometriosis and adverse obstetric outcomes (pregnancy, delivery, and neonatal). The odds ratio (OR) and 95% confidence interval (CI) were calculated after adjusting several intervening variables such as age, etc. The level of significance was set at 0.05, and all results were expressed as absolute and percent frequencies for qualitative variables or as the mean ± SE for quantitative variables. All data were analyzed with Statistical Package for the Social Sciences, version 17.0, SPSS Inc., Chicago, Illinois, USA (SPSS).

## 3. Results

All women in the endometriosis group had undergone endometriosis surgery, and at least one ovary had endometrioma. 68 women had colorectal endometriosis, and the treatment approach was superficial shaving or discoid resection of the rectal endometriosis to remove the deep nodules. A total of 250 participants (125 people in each group) were analyzed. Table I demonstrates the maternal characteristics in the endometriosis and control groups.

There was a statistically notable difference regarding preterm birth, placenta previa, placenta accreta, placental abruption, pre-eclampsia, gestational hypertension, GDM, and PPH in the endometriosis group compared to the control group. Among the endometriosis group, 68 women (54.4%) had rectal endometriosis. A significant difference was observed between the group with rectal endometriosis and the group without rectal endometriosis in terms of SGA (p = 0.03). In the rectal endometriosis group, pregnancy outcomes including preterm birth (p < 0.001), PPH (p < 0.001), placenta previa (p = 0.04), placenta accrete (p = 0.04) and placenta abruption (p < 0.001), pre-eclampsia (p = 0.04), and GDM (p < 0.001) increased with a significant difference compared to the control group. Also, the rate of CS in the control group was significantly higher than in the rectal endometriosis group (p < 0.001) (Table II).

In the endometriosis group, women with adenomyosis (n = 54) experienced a higher incidence of PPH compared to those with endometriosis but without adenomyosis (n = 71).

**Figure 1:**
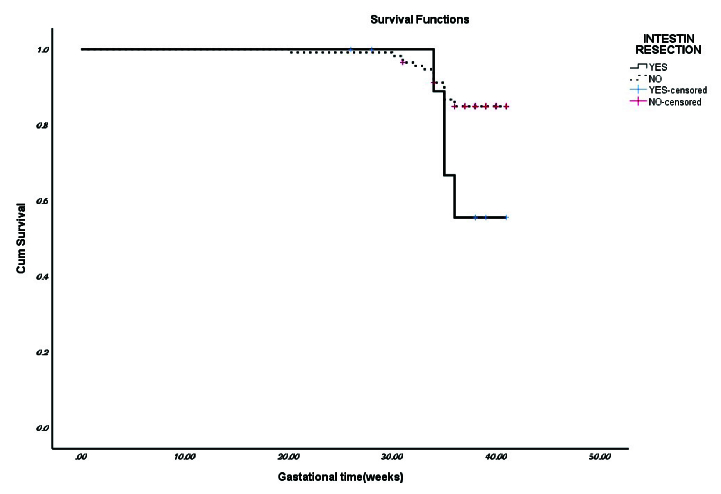
Assessment of preterm birth predictive factors.

In the assessment of premature birth predictive factors in the endometriosis group among the factors of maternal age, the presence of adenomyosis, and rectal endometriosis, only not having rectal endometriosis was a protective factor, relative risk: 0.33 (0.114–1) (Figure 1).

Table III shows neonatal consequences. NICU admission was remarkably higher in neonates of the endometriosis group than in the control group (40.7% vs. 24.8%, p = 0.009). However, no statistically significant differences were seen in the mortality rate of neonates (0.8% vs 4%, respectively, p = 0.12). None of the infants met the large for gestational age criteria at birth.

The mean and SD of the birth weight of infants in the endometriosis group was 3056 ± 68 gr and in the control group was 3061 ± 66 gr (p > 0.05).

**Table 1:** Pregnancy characteristics between groups (n** = **125)


**Maternal characteristics**	**Endometriosis group**	**Control group**	**P-value**
**Age (yr)**	32.74 ± 4.10	31.70 ± 5.53	0.94
**BMI (kg/m^2^)**	24.28 ± 3	23.49 ± 3.09	0.04
**Gravidity (n)**	1.57 ± 0.6	2.3 ± 1.2	< 0.001
**Parity (n)**	1.2 ± 0.4	3.37 ± 2.29	< 0.001
Data are presented as Mean ± SD,* t* test, BMI: Body mass index			

**Table 2:** Pregnancy outcomes in endometriosis and control groups


		**Endometriosis group**					
**Outcome**	**Control group**
		**(n = 125)**	**Total (n = 125)**	**With rectal endometriosis (n = 68)**	**Without rectal endometriosis (n = 57)**	P-value*	P-value**	P-value***
**Preterm birth < 37 w**	3 (2.4)	21 (16.8)	10 (14.7)	11 (19.3)	0.03				0.49	< 0.001
**Postpartum hemorrhage**	2 (1.6)	35 (28)	23 (33.8)	12 (21.1)	< 0.001	0.11	< 0.001
**Placenta previa**	0 (0)	4 (3.2)	3 (4.4)	1 (1.8)	0.04	0.39	0.04
**Placenta accrete**	0 (0)	6 (4.8)	3 (4.4)	3 (5.3)	0.01	0.83	0.04
**SGA**	5 (4)	10 (8)	9 (13.2)	1 (1.8)	0.22	0.03	0.23
**Pre-eclampsia/hypertension**	9 (7.2)	29 (23.2)	14 (20.6)	15 (26.3)	< 0.001	0.45	0.04
**Eclampsia **	1 (0.8)	1 (0.8)	0 (0)	0 (0)	0.99	-	0.50
**Placenta abruption**	0 (0)	25 (20)	15 (22.1)	10 (17.5)	< 0.001	0.53	< 0.001
**GDM**	4 (3.2)	13 (10.40	10 (14.7)	3 (5.3)	0.02	0.08	< 0.001
**CS **	89 (71.2)	58 (46.4)	33 (48.5)	25 (43.9)	0.4	0.54	< 0.001
**Stillbirth**	2 (1.6)	0 (0)	0 (0)	0 (0)	0.09	-	0.31
**Bowel perforation**	0 (0)	0 (0)	0 (0)	0 (0)	-	-	-
**Hemoperitoneum**	0 (0)	0 (0)	0 (0)	0(0)	-	-	-
Data are presented as n (%). Chi-square test. SGA: Small for gestational age, GDM: Gestational diabetes mellitus, CS: Cesarean section. *Between control and endometriosis group, **Between with and without rectal endometriosis, ***Between control and with rectal endometriosis group							

**Table 3:** Neonatal complication in endometriosis and control groups (n = 125)


**Neonatal outcomes**	**Endometriosis**	**Control**	**P-value**
**NICU admission**	46 (40.7)	31 (24.8)	< 0.001
**Mortality**	1 (0.8)	5 (4)	0.12
Data are presented as n (%). Chi-square test, NICU: Neonatal intensive care unit			

## 4. Discussion

The study's most important finding was that endometriosis could be considered an independent risk factor for increase in obstetrical complications. The risk of placenta previa, placental abruption, pre-eclampsia, preterm birth, GDM, and PPH in the endometriosis group was higher compared to the control group. In the endometriosis group, 54.4% of women had rectal endometriosis. The risk of SGA was significantly higher in rectal involvement. Other adverse outcomes were not associated with rectal endometriosis. Even though in our study, the rate of preterm birth in the endometriosis group was significantly higher than in the control group, the SGA rate in both groups was not significantly different, indicating no growth restriction. In the endometriosis group, women with rectal endometriosis had a higher rate of SGA, which raises the possibility that deep endometriosis was responsible for this complication. The underlying mechanisms relating to endometriosis and pregnancy complications remain primarily unclear (19).

A meta-analysis of 33 studies shows a borderline association between endometriosis and low-birth weight, similar to our results in rectal endometriosis (12). Our findings were consistent with previous studies that found an association between endometriosis and other placental complications, such as placenta previa (20–22). In our study, the risk of placenta previa was significantly higher in the endometriosis group compared to the control group. Our findings did not show a significant difference in placenta previa in the group with rectal involvement. However, out of 4 cases of placenta previa in the endometriosis group, 4 had rectal nodules. The small sample size of our study may explain the lack of significant correlation. Also, rectal endometriosis surgery or medical treatment did not change the risk of placenta previa.

The incidence of placenta previa was 7.6% in rectovaginal endometriosis, 2.1% in endometrioma plus peritoneal endometriosis, and 2.4% in peritoneal endometriosis only. The risk of placental anomaly in women with rectovaginal endometriosis was 6 times higher (23). A strong association was observed between deep endometriosis and placenta previa (20). In our study, placenta accrete significantly increased in the endometriosis group. Of 6 women with placenta accreta, 2 had a history of previous CS, and the rest were primigravida. This indicates that the cause of placenta accreta was unrelated to any previous CS history. Our findings did not demonstrate any association between rectal endometriosis and increased risk of placenta accreta. Nonetheless, a meta-analysis of 48 studies demonstrated a strong relationship between endometriosis and placenta accrete (24). Limited evidence from a few studies also indicated that surgical excision of endometriosis may not reduce the risk of adverse pregnancy outcomes (15).

Since there is no consensus on the effect of endometriosis surgery in different stages, the findings of our study demonstrated that the survival of the term pregnancy in rectal endometriosis increases the risk of preterm births.

Similar to our study, in many studies, the CS, placenta previa, gestational hypertension, and FGR rates did not increase with laparoscopic surgery for rectal endometriosis nodules. In some studies, the increased risk of CS after endometriosis rectal nodule surgery may be due to obstetric decision-making for CS, not the clinical necessity of CS (14). However, in some studies, no difference was observed in the live birth rate, clinical pregnancy rate, and adverse pregnancy outcome in rectovaginal endometriosis treated either conservatively or operatively (25). Moreover, pregnancy complications, regardless of the surgical technique of rectal endometriosis, occurred in half of the pregnancies, and no increase in complications after surgery was reported (26).

In our study, the NICU admission rate was significantly higher in the endometriosis neonates than in the control group. However, the rate of neonate mortality was not different in the 2 groups. According to a similar study, no difference was observed in neonatal outcomes between vaginal delivery and CS (27). One neonatal mortality occurred after an emergency CS for fetal distress in a woman with rectal endometriosis. The 5-min Apgar score and arterial PH were 0 and 7.07, respectively.

### Strength and limitations 

Our hospital is a tertiary referral center for endometriosis, especially severe endometriosis, and we had all phenotypes of the endometriosis group in our study. The limitations of our study include the small sample size and the retrospective nature of the study, which potentially increased the risk of missing data and selection biases. Therefore, extensive epidemiological studies in different populations (e.g., races and regions) are needed to clarify the magnitude of these risks to define the appropriate level of proactive management of pregnant women with endometriosis.

## 5. Conclusion

Women with endometriosis are at a higher risk for important adverse maternal outcomes from a clinical view, women with endometriosis should benefit from increased surveillance during pregnancy to prevent neonatal and maternal complications. Due to increased delivery complications, such as increased CS and PPH in the endometriosis group, adequate preparation during delivery, such as uterotonic agents and blood products, should be considered.

##  Data availability

Data supporting the findings of this study are available upon reasonable request from the corresponding author.

##  Author contributions

Samaneh Rokhgireh: Designed and directed the project; Neda Hashemi: Wrote the manuscript, was involved in planning, and supervised the work; Fatemeh Shahmoradi: Data collection, draft manuscript preparation; Marziyeh Noori: Data collection, draft manuscript preparation; Roya Derakhshan: Monitored, evaluated, and analyzed the result of the study. Ladan Haghighi: Wrote the manuscript and was involved in planning and supervising the work. All authors approved the final manuscript and take responsibility for the integrity of the data.

##  Conflict of Interest

The authors declare that there is no conflict of interest.
